# Breastfeeding and humanitarian emergencies: the experiences of pregnant and lactating women during the earthquake in Abruzzo, Italy

**DOI:** 10.1186/s13006-022-00483-8

**Published:** 2022-06-15

**Authors:** Angela Giusti, Francesca Marchetti, Francesca Zambri, Elide Pro, Eleonora Brillo, Sofia Colaceci

**Affiliations:** 1 National Center for Disease Prevention and Health Promotion, National Institute of Health, Rome, Italy; 2 National Institute of Health, Viale Regina Elena, 229, 00161 Rome, Italy; 3 Italian Red Cross, Milan, Italy; 4 Department of Obstetrics and Gynecology, Fabrizio Spaziani Hospital, Frosinone, Italy; 5 Department of Biomedicine and Prevention, Tor Vergata University, Rome, Italy; 6 Center for Research in Perinatal and Reproductive Medicine, University of Perugia, Perugia, Italy; 7 Saint Camillus International University of Health and Medical Sciences (UniCamillus), Rome, Italy

**Keywords:** Infant and young child feeding in emergencies (IYCF-E), Earthquakes, Pregnant women, Breastfeeding, Emergency preparedness, International code of Marketing of Breast-milk Substitutes

## Abstract

**Background:**

Emergencies have a great impact on infant and young child feeding. Despite the evidence, the recommended feeding practices are often not implemented in the emergency response, undermining infant and maternal health. The aim of this study was to explore the experiences of pregnant and lactating women during the earthquake emergency that occurred in L’Aquila on 6 April 2009.

**Methods:**

The study design was qualitative descriptive. Data were collected by individual semi-structured interviews, investigating the mother’s experiences of pregnancy, childbirth, breastfeeding, infant formula or complementary feeding during the emergency and the post emergency phase. Data analysis was categorical and was performed by using N-Vivo software.

**Results:**

Six women who were pregnant at the time of the earthquake were interviewed in January 2010. In addition to the essential needs of pregnant and lactating women, such as those related to the emergency shelters conditions, the main findings emerged from this study were: the reconfiguration of relationships and the central role of partners and family support; the need of spaces for sharing experiences and practices with other mothers; the lack of breastfeeding support after the hospital discharge; the inappropriate donations and distribution of Breast Milk Substitutes.

**Conclusions:**

During and after L’Aquila earthquake, several aspects of infant and young child feeding did not comply with standard practices and recommendations. The response system appeared not always able to address the specific needs of pregnant and lactating women. It is urgent to develop management plans, policies and procedures and provide communication, sensitization, and training on infant and young child feeding at all levels and sectors of the emergency response.

## Background

Every year hundreds of thousands of people are affected by natural or humanitarian disasters around the world. A disaster is defined as a calamitous event that leads to loss of lives, great human suffering and distress, and large-scale material damage [1]. The cause of disasters, and the related state of emergency, can be natural (earthquakes, floods, hurricanes, droughts, epidemics) or human-caused (conflicts, migrations, nuclear disasters) [2].

In Europe, in recent years, there has been an increase in the frequency and intensity of natural disasters, with dramatic consequences for populations and societies in the short, medium and long term [3, 4]. With regard to hydrological and meteorological disasters such as droughts, extreme temperatures, fires, floods and storms, this increase is closely related to climate change [3, 5, 6].

**Fig. 1:**
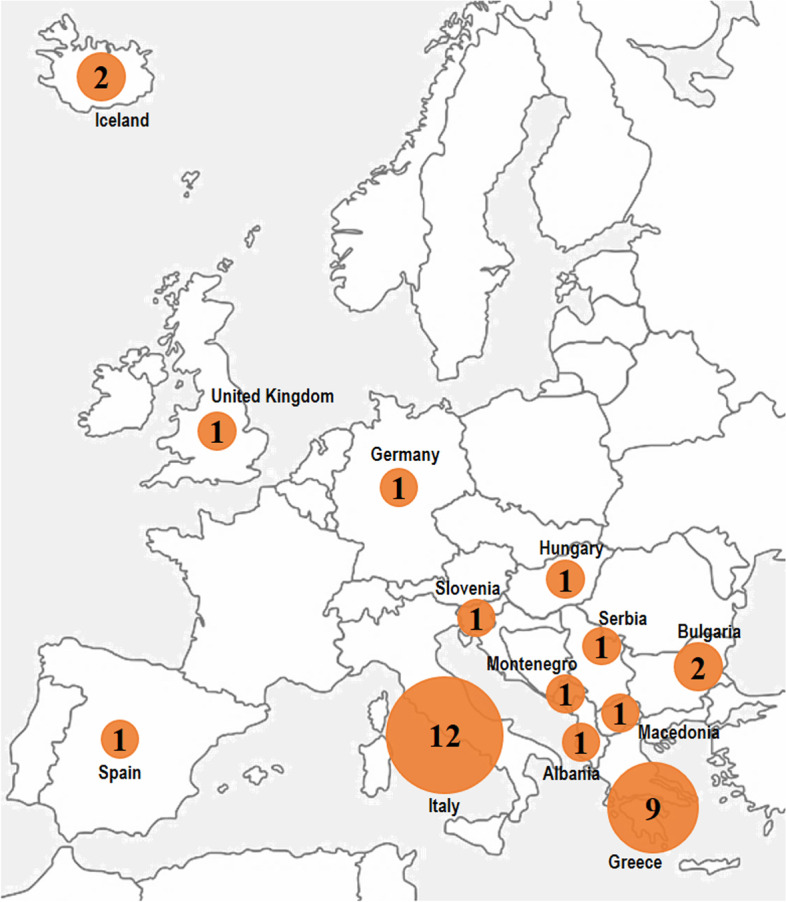
Number of reported earthquakes in Europe by country 2000-2017 [7]

Among natural disasters, earthquakes represent one of the most lethal in terms of human and economic losses, also due to their unpredictability and their devastating impact. Between 2000 and 2017, 34 earthquakes occurred in Europe (Fig. 1), affecting 257,303 people (of which 701 died) and causing economic damages for an amount of over 32 billion Euros [7]. Amongst the countries affected by earthquakes in Europe, Italy has suffered the greatest losses: between 2002 and 2016, four seismic events occurred (San Giuliano in 2002, L’Aquila in 2009, Emilia Romagna in 2012 and central Italy in 2016), producing a total of 679 deaths and 124,000 injured [7]. Italy, due to its particular geodynamic context, is a country characterized by a high seismic hazard, as 37.6% of Italian municipalities fall into the two higher classes of earthquake hazard; moreover, the country presents a considerable hydrogeological, volcanic, seaquake and fire risk [8–10].

All types of emergencies have a significant impact on the health of the population. Elderly people, people with disabilities, chronic conditions or other pathologies, pregnant women, infants and young children, are particularly vulnerable and take a high share of the disease burden associated with emergencies [11]. In addition to the disaster-related urgent needs, such as safety, shelter, food and clean water, infants (0-12 months), young children (< 2 years) and pregnant women have specific needs that require an immediate and adequate response [12].

According to national and international recommendations [13–17] there are some priority actions to be taken with regard to Infant and Young Child Feeding in Emergencies (IYCF-E) (Table 1). In these situations, it is necessary to protect, promote, and support breastfeeding and infant and young child feeding optimal practices, improving the early initiation of exclusive breastfeeding in all newborns, limiting Breast Milk Substitutes (BMS) supplementations as much as possible, and including, among the key newborn health interventions, skin-to-skin contact, kangaroo mother care, delayed umbilical cord clamping, and rooming-in [17]. The World Health Assembly urges Member States to ensure that national and international preparedness plans and emergency responses follow the evidence-based Operational Guidance on Infant and Young Child Feeding in Emergencies [17], to address the specific needs of pregnant or lactating women and infants 0-2 years old, breastfed and not breastfed. This implies that health professionals and first aid volunteers should be trained in relation to these specific populations [17].

**Table 1:** Six practical steps for emergency preparedness [17]

1. Endorse or develop policies	
2. Train staff	
3. Coordinate operations	
4. Assess and monitor	
5. Protect, promote and support optimal infant and young child feeding with integrated multi-sector interventions	
6. Minimize the risks of artificial feeding	

In Italy, in recent years there has been a raising attention to the implementation of emergency preparedness systems focusing on infant and young child feeding [18–21]. On 6 April 6 2009 an earthquake of magnitude Mw 6.3 struck the town and province of L’Aquila, causing widespread damage. Because of the disaster, about 100,000 buildings were damaged, 1600 people remained injured, 309 deceased, and 66,000 people were displaced [22]. This study was conducted after the earthquake by the AINE (Alimentazione Infantile Nelle Emergenze – Infant Feeding in Emergencies) working group, consisting of national experts in breastfeeding protection, promotion and support in emergencies. In its primary intention, the research was designed to inform local action on mothers and infants’ care. Twelve years later the AINE working group is still operational and is dealing with the COVID-19 pandemic. This latter has re-raised the issue of the limited availability of studies exploring the emergency responses from the mothers’ perspective [23], and the ongoing obstacles and challenges affecting IYCF-E worldwide [24–29]. Therefore, the authors decided to re-analyze the data collected during the earthquake in Abruzzo with a focus on the recommended practices for breastfeeding protection, promotion and support. The aim of this study, thus, was to explore women’s experiences of pregnancy, childbirth, and infant feeding during and after L’Aquila earthquake emergency. Moreover, we examined how health care system and emergency response system were able to address their expressed needs. Although the article has a focus on the status of breastfeeding and IYCF-E protection, promotion and support, other aspects of pregnancy and infant care that emerged from the study were described.

## Methods

The study design was qualitative descriptive, according to the COREQ checklist [30], with a phenomenological approach. The purposeful sampling included women who were pregnant and lived in the affected area at the time of the earthquake. The women were contacted through the health care services provided in the camps and the emergency network; all accepted to participate and signed an informed consent. The data collection was performed by individual semi-structured interview (30-60 minutes); this methodology was chosen due to the precariousness of housing and the geographical displacement of women.

The interviews were conducted by a study researcher and included a general question on the mother’s experience of pregnancy, childbirth, breastfeeding, infant formula or complementary feeding during the emergency and the post emergency phase. In-depth information was sought through probing, with regard to essential needs (e.g. housing, food, safety), everyday life and health care provision, exploring the areas of strength, the challenges and the current needs (Table 2). Each participant reported socio-demographic data on an anonymous, self-administered form. The interviews were conducted at the women’s home or in their temporary accomodation, on their invitation. All the interviews were digitally audio-recorded and fully transcribed Table 3.

**Table 2:** Semi-structured interview questions

What has been your maternity experience (pregnancy, childbirth and infant feeding) during the emergency?	
*Probes:*	
- What difficulties did you encountered?	
- What helped you in these situations?	
- How do you feel now? What are your current needs?	
- What are your suggestions to improve the well-being of mothers, babies and families in emergency situation?

**Table 3:** Themes, categories and codes

Theme	Category	Code
Essential needs, basic services and security	Women’s feelings	- Fear of seismic shocks - Fear for the safety of the fetus - Need for stability and “return to normality”
	Shelters and temporary accommodations	- Characteristics and conditions - Keeping the woman close to her partner - Keeping the woman close to a maternity facility
Community, partner and family support	Reconfiguration of relations	- Separation from partners - Support from the family - Positive effects of the relationship with the baby
	Community	- Absence of the community to which women belong - Social pressure - Families perception of being a burden for the emergency response system
Mother-Infant focused, non-specialized support	Emergency personnel	- First aid personnel - Health and psychosocial professionals
	Emergency services and initiatives	- Aimed to family support - Aimed to promote the psycho-social health - Meeting areas for mothers and children
Specialized Maternal and Infant health care	Hospital care	- Welcoming care - Acknowledging the specific needs due to emergency - Feeling of being taken care of, together with the family - Sharing the birth experience with other pregnant women - “Old-fashion” breastfeeding hospital practices
	Community services	- Breastfeeding support after hospital discharge - Standard health care
	IYCF-E	- Infant’s products and infant formula distribution - Support and education on BMS reconstitution - Infant formula supplies

*Abbreviations:* *IYCF-E* Infant and Young Child Feeding in Emergencies, *BMS* Breast Milk Substitutes

Analysis was conducted following a secondary analysis approach (SDA) [31]. Data analysis was categorical: categories were developed both deductively, based on the research question, and inductively, based on emerging contents. The SDA was conducted on the original fully transcribed interviews by two researchers from the original team (AG and EP) and by new researchers who subsequently joined the research team (FM, FZ, SC, EB). Reflexivity was addressed during the SDA through meetings involving the research team. The analysis was performed coding all the transcripts by using N-Vivo software.

**Fig. 2:**
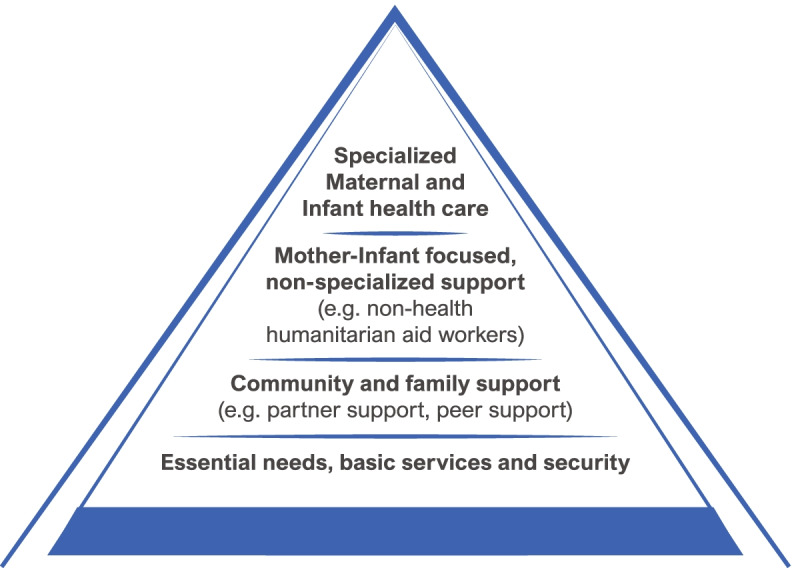
Intervention pyramid for mental health and psychological support in emergencies [32] adapted to Maternal and Infant Health

The emerging women’s need and care provided were classified in main themes, according to the Guidelines on Mental Health and Psychosocial Support in Emergency Settings [32], adapted to maternal and infant care (Fig. 2).

## Results

Six women who were pregnant at the time of the earthquake (6 April 2009) were contacted in January 2010, 8-9 months after the emergency. They all lived in one of the municipalities of L’Aquila province; they were affected by the earthquake and were forced to leave their homes.

At the time of the earthquake, the women’s mean age was 33.6 years (28-46). Four women were primigravidae, one was secundigravidae and one tertigravidae. The gestational age was 6 to 40 weeks. Three women had a spontaneous vaginal birth, while three had a cesarean section. Three infants were exclusively breastfed, while three were using/had used infant formula.

At the time of the interview, three women had returned to their homes, scarcely compromised by the earthquake, and declared safe. The others were still hosted in hotels, waiting for a more stable accommodation assignment. The babies had an age ranging from 1 to 9 months of life. 

The following themes, categories and codes (Table 3) were identified with the categorical analysis of the data.

### Essential needs, basic services and security

Concerning the direct experience of the earthquake and the response to essential needs, the trauma and the fear of seismic shocks were common to all the interviews and constant over time, in the immediacy of the events and at the time of the interview:

*“ [ …] On the night of the earthquake we woke up with a big rumbling sound inside the house, the walls swaying ... my husband went to the children’s room, took them and went out, I took the blanket from our bed and followed them. We were very scared.”*

Some pregnant women have also reported that the safety of their fetus was the main concern at the time of the seismic event:

*“The earthquake was a trauma, everything was shaking and I was just thinking: I hope it will end soon! We rushed away immediately, me, my husband and my mother-in-law, who lives with us. It was terrible: half of the house collapsed. But the thing that scared me the most was to know if the baby was okay.”*

Living in temporary housing was very hard for many women. Their accommodation initially consisted of makeshift housing (e.g. cars, caravans), then emergency camp tents. Only two of them moved away from the earthquake-stricken areas immediately after the earthquake, while the other four spent between 1 and 3 days in their car before moving into the tents, reporting a considerable discomfort.

*“ … then in the evening, at a certain time, we went to the farmyard [ …] with the car turned on all night. Well, it was quite a bit uncomfortable, because with my belly ... she [3-years-old daughter] was sleeping behind: we couldn’t lay down all the seats. Anyway, at night, I had to get up four or five times to pee, go out, and it was so cold in the yard! What could we do? And after four or five days they came to set up the tents.”*

In the weeks after the earthquake, the accommodation varied according to the availability, i.e. their homes (when considered safe), local hotels, hotels in a different location, or hosting by relatives. In some cases, a better accommodation option has been offered, but was declined, as it would have involved separating the family. The time spent in the tents was variable, from a few days to a month and a half. These temporary shelters caused considerable discomfort to the pregnant women, determined by the cold climate inside the tents and the impossibility to rest, especially in case of threat of preterm labour, and, above all, by the absence of dedicated or adequate toilets (poor hygiene, lack of privacy, location outside the tents):

*“And then the worst thing: the toilets. Staying in that space where everyone goes and so you can't even lean on, because obviously they were filthy. My husband had to hold my hands like that, because I couldn't stand it anymore. How many times did I hold back the pee to not wake him! Because it was raining outside … but I could not go alone. I think there was a need for a bathroom only for pregnant women.”*

*“I couldn't get in because I had such a big belly that I couldn't get into it.”*

*“I always had a sore throat, in short, always a little cold ... because it was cold, the tent was damp, the heating was not enough.”*

During the first days of emergency, in the temporary housing, the interviewees suffered deprivation of privacy and difficulties related to living with many other people, sometimes strangers. In some areas, pregnant women and their relatives were provided with private tents. One woman experienced the family tent and reported high satisfaction. Because of the discomfort caused by the everyday life in the camps, some of the women and their partners took action to find another accommodation that was suitable for their needs.

In general, the time taken by the institutions to allocate housing other than tents was not adequate to promptly meet women’s need to reprogram the place of birth and find an accommodation close to it. Therefore, most couples tried to find a solution that could meet at least two main needs: to keep the woman close to her partner, who had resumed work immediately after the earthquake, and to be close enough to a maternity facility, safe from the seismic shocks. This resulted in repeated moving that, for some women, were added to other temporary moving determined by the hospital admission or other health care checks.

*“So, in mid-May, they sent us to the hotel. But we moved twice. The first to P.* [a town where there were some hotels that had been made available for the earthquake victims]*, but I was a bit far from S.* [the municipality of the hospital where the woman had decided to give birth]*. So I asked for another hotel closer to S. […].”*

*“In May I went to P. and I stayed there for a month. Then, from May to October [...] I was on the coast. In November I returned to L.. My husband bought a caravan, to stay closer to the hospital: I had to do medical checks and I wanted to give birth in L..”*

*“So I went first to my sister-in-law’s house and then, at the end of May, I went to the coast. My husband was not always with me, he came and went. [...] My husband worked, so he settled in some friends' house* [in the earthquake area] *and I was on the coast with my mother-in-law. He used to come every now and then.”*

Women also expressed a need for a place and space close to their families to care at the same time for the baby and their older children.

After childbirth, the women who were forced to change the accommodation several times, expressed a strong need for stability and “return to normality”. This referred to more stable place, next to their village, their house, their belongings, affection, relationships, social life and memories and the opportunity to return to work.

*“Normality. Here we feel very fine, it’s a lovely village, because I love it, I didn’t expect it, we really feel good. But it’s not your village, you do not have your home.”*

*“A stable place, from which we no longer have to move [...]. Something we can say, for now: - This is home! -. Because here we live with our suitcases, the suitcases must be enough for everything. Because you never know when you’ll have to leave, you don't have all your stuff. My clothes are limited, all limited, all limited.”*

### Community, partner and family support

During the emergency, the women experienced a noticeable reconfiguration of relations, characterized by a reduction in the time spent daily with their partner, and by an increase, in some cases, of relationships with other family members or forced cohabitation with people not belonging to the family. Being displaced far from their homes or villages, many of them suffered the absence of the community they belong to and the consequent lack of support from their relatives and friends, especially after childbirth and for the care of the baby and the siblings.

Again, the separation from partners was reported as the greatest challenge:

*“The hardest thing for me was being away from my husband. [...] Whatever could have happened, and he was not there: I was always alone. Of course, with my mother, my sister ... but not him! We haven’t lived the experience together…”*

*“We didn’t have any deaths in the family, but we felt really alone: me on one side and my husband on the other. We used to cry so much by phone, I think the child has suffered, I was really sad!”*

*“For a while he was with me (...) then he had to come back: he was traveling back and forth, returning on Friday or Saturday. But for me it was horrible, because ... alone with the two of them ... [the baby and the older sister].”*

For some women, the support from the family was significant, as was the relationship with their baby, which represented an important element of well-being:

*“Back at the hotel, my mother-in-law helped me a lot, she made me eat even if, often, I didn't want to. She said to me: "You have to try hard." And then I saw that the baby was fine. The pediatrician said: "He grows well, keep it up!" And so, I'm doing it this way ... I always keep him next to me, even at night… he stays with me and my husband, when he comes back ... I feel safer having him [the baby] next to me. Besides, I really like that baby smell...”*

On the other hand, a mother told how negative the social pressure focused on child care and breastfeeding was, in a moment of intense vulnerability and emotional shock in which she needed to focus on her own needs and resources:

*“The milk? The people and even my mother-in-law [they said]: - No, you have to breastfeed, you have to force yourself, you have to believe in it, you have to do it. - That fact... that made me feel even guilty, do you understand? Okay, I didn’t feel like doing it! For me it was easier to give him the bottle. Even at night, I couldn’t even wake up. Although, fortunately, he always slept: he woke up to eat, I put him back to sleep and he slept. He woke up two or three times ... for me it was a burden... I couldn’t do it ... Apart from sleep, I just refused it ... but then ... You know that breast milk is good, and so you had that contrast of thinking: - No, it’s necessary! - …But I wanted to give him the bottle, even to try to make him sleep longer at night!”*

The women reported the feeling of having been abandoned by the institutions. They felt that the Municipality failed to address their housing needs:

*“It was bad (…) we went to the Mayor, but we had no answer. They still didn’t handle the situation”*

*“We received more from strangers than from ... you see… we felt a little abandoned, [..] we thought the Municipality would be closer to us.”*

Furthermore, women had the perception that, in the post-emergency management, families with pregnant women, babies or young children were considered a burden:

*“We were a burden, because one who says to you – Don’t make me think about it too, go to the coast! [the accommodation provided on the coast]- It means that you are a burden!”*

### Mother-infant focused, non-specialized support

The first aid personnel were reported with gratitude as being the “*angels in uniform*”. The health and psychosocial professionals were a relevant presence for the psychological well-being of mothers, as well as the services aimed to family support (i.e. campus for children). Instead, the professional’s turnover and the consequent loss of meaningful nodes within the emerging support network was reported as highly frustrating. Due both to the population’s displacement and to the staff turnover, the meeting areas for mothers and children were available in very rare cases but, where present, they were considered useful:

*“They wanted to organize meetings with mothers and children, even just mothers to do activities together a few times a week. Having a chance to hang out would have been nice, I think.”*

Some initiatives aimed to promote the psycho-social health of women and parents with young children were reported, as the implementation of dedicated spaces for older children. On the other side, the system failed in providing occasions for group support (peer-to-peer support, mutual aid, shared time, care or activities). Some women reported that it would have been useful to provide a baby-sitting service to support mothers in managing care and everyday life.

### Specialized maternal and infant health care

For some women, the birth of their baby compensated for the lack of housing, relationships and social support. The positive experience of childbirth was more evident in those hospitals that provided welcoming care to women and their partners, acknowledging their specific needs related to the earthquake experience. In these cases the hospital was perceived as a safe, protected and clean place where women could take refuge after months of unsatisfied basic needs. During the hospitalization, some women felt, for the first time since the earthquake, the feeling of being taken care of together with their family. Hospitality and emotional-psychological support provided by the healthcare personnel, together with the opportunity to share with other pregnant women and new mothers, made childbirth a moment of reassurance and reconciliation.

In some cases, women reported the support they received for the positioning and attachment of their babies to the breast after birth:

*“They attached him immediately [at my breast], more or less not even a couple of hours”.*

*“Already in the hospital they made me attach him [at breast] often.”*

Specifically a woman whose daughter was hospitalized a week after birth spoke about the breastfeeding support she received in the hospital:

*“They helped me, they helped me a lot, because I wasn’t so skilled. [...] [Latching on required] a lot of patience and they helped me. They expressed my milk, considering that at midnight they sent me away [from the Neonatal Intensive Care Unit], they gave it [to the baby] and showed me how I should do [...]: - Insist, attach her, even if you feel that she doesn't suck. And in fact now, at eight months, she takes only my milk.”*

On the other hand, two mothers were advised to use infant formula during the hospital stay and, in one case, a specific brand was prescribed:

*“No, I was not able, I had very little breastmilk. I was expressing it all, but the maximum I expressed was 20 ml. And so ... he had an excess of hunger. [...] He started with [brand of the infant formula], [...] [while] to the premature babies they gave [brand of infant formula].”*

*“The hospital… [recommended me infant formula] my breastmilk was not enough for him. [...] He wanted to eat every hour, he was always attached.”*

In some hospitals there was no rooming-in:

*“He seemed quieter, they told me he was resting [...]. Then when he woke up they brought it back to me.”*

The support to breastfeeding provided after discharge was insufficient both by local health services and emergency staff, with some variability related to specific services or professionals.

*“I left the hospital on Thursday, and on Saturday he didn’t pee or wake up to eat. [...] He was weakening, I immediately called the pediatrician and she told me: - Give him the infant formula -. And… nothing [...] I don’t know if he was not able to suckle at the breast anymore. For half a month I tried both [breastmilk and infant formula], but at the breast he got little or nothing.”*

*“Alone. I did it alone [once discharged from the hospital].”*

*“No, [they didn’t give me information on breastfeeding], [the pediatrician at the clinic] saw that I was breastfeeding and that he was growing well. [...] [She told me] only: - Don’t eat legumes. [...] Eat everything but not the beans, otherwise the abdominal cramps can come - but otherwise she didn’t tell me anything at all.”*

Standard health care was perceived as present and efficient:

*“The pediatrician has always been there. And even though she was also a victim of the earthquake, she was never absent in [name of the municipality], she works in several Municipalities ... The Health District almost immediately restarted its activity, in short, the doctor was there too, so let’s say that it was fine, if we needed anything there was no problem.”*

Infant’s products, included infant formula, were actively distributed into the camps to pregnant and lactating women, even without a specific clinic indication:

*“When [the relief workers] brought us all that stuff. It was really a godsend. For everything: infant formula, diapers (…), baby food, ointments that I still have, baby wipes… therefore, it was money saving.”*

*“I did not use much infant formula, I mean that we tried the different infant formula brands that they gave to me. I brought the surplus to the pediatrician, and she gave it to other babies, so I didn’t waste anything. Jarred baby food too, I brought everything [to the pediatrician]: teats, bottles, pacifiers… in short, I received a lot of things, but… how many pacifiers do I need? How many bottles? And so I brought everything to the pediatrician.”*

*“There was a man, he took special care of us (…). And he brought me a lot of stuff, always from the [reference Agency of the area]: infant formula, stuffed toys, bottles, pacifiers, everything, everything, everything! I didn’t even know where to put it! We had to come and put it here in the garage! But, besides me, they went to the tents: they brought [these goods] to the other pregnant women too. So, I mean, for that [aspect] the care was great, immediate, they said – If you need something, come down and ask us.”*

It appears, from mothers’ narratives, that initial assessment of need for infant formula, support and education on the correct use and reconstitution of BMS were not provided to the parents that had received donations of infant formula or undertaken for other reasons this type of feeding for their babies. The supplies of infant formula have not been granted throughout the emergency phase, especially when the infant formula prescribed by the pediatrician was not available in the donation stock. One mother that was discharged from hospital with her healthy newborn on ready-to-use infant formula, referred major supplying difficulties, as it was not available in the local shops:

*“I’m going to [main town, distance 100 km] to buy it, because the shops that are here… despite the fact that they have it… given how much he [the baby] used to eat, I needed higher volumes, and they don’t want to bring it to me.”*

The two women who had interrupted breastfeeding were asked if anyone ever proposed them the relactation, but both answered negatively.

Women reported difficulties in feeding and caring for their baby. In some cases breastfeeding was perceived as a burden, and consequently interrupted and substituted with infant formula feeding; in others, infant formula was prescribed and this was perceived as frustrating by those mothers that would have otherwise preferred to breastfeed. One woman expressed a great unease in caring for her newborn and young child and a great sense of isolation, highlighting the lack of support services.

## Discussion

Although the essential needs of pregnant and breastfeeding women are similar to those of the general population (food, shelter, water, basic healthcare, infection control, and safety), they have some specificity that must be taken into account when addressing an emergency. The available literature highlights how natural disasters impact psychologically on pregnant women, which can develop short and long term traumas, often resulting in increased levels of stress and anxiety [32, 33]. During the emergency, one of the main concerns of pregnant women, as confirmed by our study, is the safety and the well-being of their unborn baby [23, 34–36]. Other main concerns are the challenges related to the loss of one’s home, the displacement, and the move to other temporary housing solutions. In these situations, pregnant and lactating women face difficulties that relate specifically to their condition [35]. The emergency shelters do not always guarantee spaces suitable for physical needs (dedicated toilets, warm environments) and the need for privacy and intimacy of women and families. All of this can lead to a further increase in psychological distress, as resulting also for the general population [37].

In addition to the physical environment, natural disasters deeply impact on relationships [38]. Our study shows the strong need to protect meaningful relationships and social networks (partner, relatives, friends, neighborhood, and community). Social support plays a key role in traumatic and emergency situation to promote the well-being, quality of life, and resilience of the affected population [32, 38–40] and are considered a potential resource for relieving depression in pregnant women [41]. Over the L’Aquila earthquake experience, family was an extremely important support system for emergency and post-emergency everyday life, and women clearly expressed the need to be close to their partners. Therefore, emergency response has to support this social unit [38], as a means to promote people’s health and wellbeing, e.g. by designing emergency systems that allow families to remain united [32].

This study also highlights the importance of offering services that encourage the encounter with other mothers and children, in peer groups. As reported by Sezgin & Punamäki [40], peer and neighbor relations can represent a valid aid in sharing the disaster experience, as well as in the practical management of children. Moreover, providing opportunities for interaction between families could relieve the sense of isolation and mitigate the loss of the belonging community. On this concern, it could be opportune to identify and involve mother-to-mother breastfeeding support organizations, such as La Leche League [17].

One of our interviewees reported great difficulties, highlighting the burden of taking care alone of her young children, the sense of inadequacy, and the perception of social pressure and judgment. In emergencies, traumatic experiences and challenges, included the separations from partners and relatives, add to the new mother’s post-partum peculiar difficulties, increasing the risk of adverse mental health effects [42], such as postpartum depression and Post Traumatic Stress Disorder [43]. As recommended by IASC guidelines [32], in the emergency response it is necessary to individuate people who require more focused psychosocial interventions.

As for specialized maternal and infant care, most of the women interviewed in this study reported a positive childbirth experience and the feeling of being welcomed by the maternity services. The satisfaction of the women was mainly due to the helpfulness and kindness of the health personnel and to the personalization of the care [44] received as victims of the earthquake. Greater obstacles emerged, instead, regarding infant feeding during the hospital stay. The hospital practices described by women (e.g. infant formula prescription in the absence of clinical indications, no rooming in, newborns fed on a schedule) were not in line with the recommended standards [12, 17].

Some women perceived that health personnel had adequately supported them during their hospital stay, especially in the very first hours after birth. In this regard, more than one of them reported how the health personnel *“attached the baby to the breast”*. This approach was widespread in Italy in the last decades, when “positioning a baby at the breast” was considered a professional competence, according to the educational programs available at that time [45]. More recently, in light of the evolving evidence, a different paradigm has emerged, considering the newborn and the mother’s competencies and relationship as the core for breastfeeding success [46–48]. In this vision, the mother-newborn dyad should be supported in “doing it themselves”, with the goal being to create an environment that allows instinctive behaviour that facilitate breastfeeding to be expressed without interference [49].

Appropriate, evidence-based, and timely support of IYCF-E saves lives and protects child health and benefits mothers and families [15, 17]. As stated by Gribble et al. [50], in emergency conditions targeted support for the caregivers of both breastfed and formula fed infants is crucial for continuing breastfeeding despite the challenges accessing the resources needed to safely formula feed. In our study, breastfeeding support after discharge at community level appeared to be inhomogeneous, despite standard healthcare was often available. Mothers’ narratives revealed a lack of breastfeeding support, resulting, in some cases, in difficult experiences, inappropriate infant formula prescription, or early disruption of breastfeeding. In emergencies, infant formula should be limited to specific conditions, including the mother’s decision, following an individual assessment performed by skilled professionals. This has to consider other cultural appropriate strategies (e.g. relactation, wet nursing and use of donor human milk), and include continuous support and educational strategies for mothers and caregivers [17]. When properly prescribed or used, infant formula should be provided for as long as the baby needs it [17]; our study highlighted the importance of preserving, where possible, the type or brand of infant formula previously used, to support the psychological continuity with pre-emergency life. Despite the recommendations [17, 51], the mothers in our study did not receive education or demonstrations on formula safe use and reconstitution, nor information or proposals for relactation.

Among the factors that explain the lack of infant feeding support after the hospital discharge, in the aftermath of earthquake, several organizational difficulties affected the healthcare provision at community as well as emergency response system level, e.g. the lack of involvement of skilled professionals and experts in IYCF-E, mother-to-mother groups and peer support associations [17].

From this study emerged the theme of inappropriate donations during the emergency response. As has happened in other contexts and emergencies [25], infant’s products, infant formula and commercially produced complementary foods were actively and widely distributed to mothers and pregnant women after the earthquake, without any prior needs assessment. This lack of prevention or management of donations was in contrast with international recommendations [15, 17, 52]. It was associated to a lack of awareness about the risks of this practice both by the mothers (that perceived it as part of an efficient care) and by the emergency volunteers, camp managers and operators. It cannot be excluded that, in some cases, uncontrolled distribution of infant products undermined recommended IYCF-E practices and breastfeeding continuation during L’Aquila earthquake emergency, although the extent of its impact was not quantified, as infant feeding variables are not included in the emergency monitoring or surveillance systems.

In the last decade several emergencies occurred in Italy, including earthquakes (Emilia-Romagna Region, Central Italy), floods, other disasters (Genoa Morandi bridge collapse), refugee and migration crisis (e.g. Balcanic route, Mediterranean Sea) and, in recent times, the COVID-19 pandemic. While the Civil Protection and the emergency systems at regional and local level (e.g. Red Cross, Non-Governmental Organizations [NGOs]) have significantly improved their response capacity [53], little or nothing has changed in regards to protection, promotion and support of IYCF-E. During the COVID-19 emergency the Italian National Institute of Health has received reports of Food Aids targeting pregnant women, mothers and babies containing infant formula as a standard provision. Years pass by, challenges do not; therefore the findings of this research remain relevant.

In ordinary non-emergency scenarios, some criticalities such as context inequalities [54], contradictory information on infant feeding [55], specific formula marketing strategies [56], and training gaps by health professionals [57] undermine the protection, promotion and support of breastfeeding. These issues may easily exacerbate in humanitarian emergency settings. Dealing effectively with infant feeding in emergency means acting in preparedness. In order to guarantee an appropriate and timely support to mothers, families, infants and young children during the emergency response [17], it is critical to develop in ordinary time a structured and multisectoral planning involving IYCF support strategies [18] and to implement specific professional training [15, 17, 24].

Our findings and the ongoing experience of AINE working group regarding COVID-19 pandemic underline that it’s necessary to raise the priority of IYCF-E into the agenda of emergency preparedness. There is a urgent need of a coordinated action targeting decision makers, health professionals, camp managers, NGOs volunteers and lay support personnel and other relevant stakeholders [58–60]. For example, training on IYCF-E should be actively offered to this population group, considering that health professionals themselves don’t contemplate the International Code on the Marketing of Breast Milk Substitutes among the main training needs [57].

Among the limitation of this study, the small sample size was due to the precarious conditions of the emergency context and the geographical displacement and isolation of the mothers. For the same reason, data saturation was not seeked. Furthermore, this study was carried out by including only mothers in the study sample. For future studies, it would be important to include fathers/partners as well, for the perspective they are carriers of [61].

Currently, AINE working group is conducting a similar study involving pregnant and lactating women during the COVID-19 pandemic. It will be useful to compare the results deriving from two different scenarios of emergency. In fact, further research is needed on this topic to better tailor national policies and programs on infant feeding during emergencies.

## Conclusions

This descriptive study contributes to draw attention to the specific needs of mothers, infant, and young children in emergencies. During and after L’Aquila earthquake, the response system appeared not always able to address the specific needs of this population groups (Table 4). These results confirm that, if breastfeeding protection, promotion and support practices are adequate in ordinary conditions, this appropriateness reflects in the whole emergency management. On the contrary, where obsolete practices, unsupported by scientific evidence, prevails, this negatively impact IYCF-E. Donations of baby food, feeding equipment and BMS require a strict management at all levels of the emergency response and is a great issue, potentially undermining safety and health of infants and mothers. To meet mothers’ and infant’s needs in the emergencies it is urgent to develop management plans, policies and procedures and provide communication, sensitization, and training at all levels and sectors, to support IYCF-E. It is therefore vital acting in preparedness and ensuring the awareness of policy and decision-makers, and programmers.

**Table 4:** Challenges and responses

Challenges	Response undertaken	Response gaps
Physical environment: - Emergency shelters characteristics and conditions - Loss of home, displacement, move to temporary housing solutions	- Supply of camp tents - Temporary optimal accommodations (hotels, homes, relative’s home)	- Suboptimal shelter conditions (lack of privacy and suitable spaces for families with infants) - Hotel accommodation sometimes distant from relatives
Psychological distress and risk of adverse mental health effects (postpartum depression, PTSD)	- When possible (e.g. security of the families’ house) keep the family together - Professional psychological and health support	- Lack of peer support groups - In several cases, the system failed in finding solutions to keep the family members together (e.g. father working far from the family accommodation)
Infant and Young Child Feeding	- Hospital welcoming - Breastfeeding support during hospital stay - Professional breastfeeding support at community level, although inhomogeneous	- Pre-emergency suboptimal feeding hospital practices (inappropriate infant formula prescription, no rooming in, newborns fed on a schedule) - Lack of widespread community/professional breastfeeding support after discharge - Inappropriate donations of BMS

*Abbreviations:* *PTSD* Post Traumatic Stress Disorder, *BMS* Breast Milk Substitutes

## Data Availability

Not applicable.

## Abbreviations

BMS: Breast Milk Substitutes
IASC: Inter-Agency Standing Committee
IFE Core Group: Infant Feeding in Emergency Core Group
IYCF-E: Infant and Young Child Feeding in Emergency
NGOs: Non Governmental Organizations
